# Improving growth properties of *Corynebacterium glutamicum* by implementing an iron‐responsive protocatechuate biosynthesis

**DOI:** 10.1111/1751-7915.14244

**Published:** 2023-03-11

**Authors:** Felix Thoma, Christof Appel, Dominik Russ, Janine Huber, Felix Werner, Bastian Blombach

**Affiliations:** ^1^ Microbial Biotechnology, Campus Straubing for Biotechnology and Sustainability Technical University of Munich Straubing Germany; ^2^ SynBiofoundry@TUM Technical University of Munich Straubing Germany

## Abstract

*Corynebacterium glutamicum* experiences a transient iron limitation during growth in minimal medium, which can be compensated by the external supplementation of protocatechuic acid (PCA). Although *C. glutamicum* is genetically equipped to form PCA from the intermediate 3‐dehydroshikimate catalysed by 3‐dehydroshikimate dehydratase (encoded by *qsuB*), PCA synthesis is not part of the native iron‐responsive regulon. To obtain a strain with improved iron availability even in the absence of the expensive supplement PCA, we re‐wired the transcriptional regulation of the *qsuB* gene and modified PCA biosynthesis and degradation. Therefore, we ushered *qsuB* expression into the iron‐responsive DtxR regulon by replacing the native promoter of the *qsuB* gene by the promoter P_ripA_ and introduced a second copy of the P_ripA_‐*qsuB* cassette into the genome of *C. glutamicum*. Reduction of the degradation was achieved by mitigating expression of the *pcaG* and *pcaH* genes through a start codon exchange. The final strain *C. glutamicum* IRON+ showed in the absence of PCA a significantly increased intracellular Fe^2+^ availability, exhibited improved growth properties on glucose and acetate, retained a wild type‐like biomass yield but did not accumulate PCA in the supernatant. For the cultivation in minimal medium *C. glutamicum* IRON+ represents a useful platform strain that reveals beneficial growth properties on different carbon sources without affecting the biomass yield and overcomes the need of PCA supplementation.

## Graphical abstract

*Corynebacterium glutamicum* experiences a transient iron limitation during growth in minimal medium, which can be compensated by the external supplementation of protocatechuic acid (PCA). Although *C. glutamicum* is genetically equipped to form PCA, PCA synthesis is not part of the native iron‐responsive regulon. To obtain a strain with improved iron availability even in the absence of the expensive supplement PCA, we re‐wired the transcriptional regulation of the qsuB gene and modified PCA biosynthesis and degradation. The final strain *C. glutamicum* IRON+ showed in the absence of PCA a significantly increased intracellular Fe2+ availability, exhibited improved growth properties on glucose and acetate, retained a wild type‐like biomass yield but did not accumulate PCA in the supernatant. For the cultivation in minimal medium *C. glutamicum* IRON+ represents a useful platform strain that reveals beneficial growth properties on different carbon sources without affecting the biomass yield and overcomes the need of PCA supplementation.

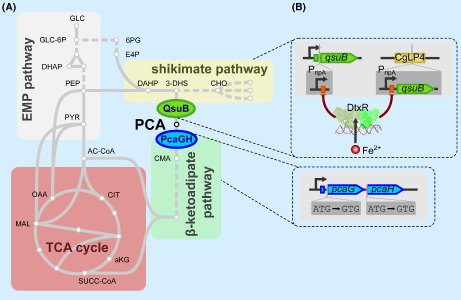

## INTRODUCTION

*Corynebacterium glutamicum* is a non‐pathogenic and facultatively anaerobic soil bacterium, which was originally isolated in 1957 as a natural l‐glutamate producer (Kinoshita et al., 1957). Nowadays it is an established workhorse in industrial biotechnology for the large‐scale production of several amino acids such as l‐lysine and l‐glutamate in million ton scale per year (Becker et al., 2018; Ikeda & Takeno, 2013; Wendisch, 2020). The high robustness, the versatile metabolism and the GRAS status are beneficial characteristics of this Gram‐positive bacterium for the application in an industrial environment. A reliable genetic engineering toolbox to tailor the metabolism is available and has been applied to expand the product spectrum of *C. glutamicum* towards various amino acids, alcohols, aldehydes, diamines, organic acids, aromatic compounds and others (Becker et al., 2018; Kogure & Inui, 2018; Wang et al., 2020; Wendisch et al., 2018; Wieschalka et al., 2013). The wild type (WT) of *C. glutamicum* is not prototrophic and growth in minimal medium essentially relies on supplementation of the vitamin biotin (Peters‐Wendisch et al., 2014). Although not of vital importance, the addition of small amounts of iron chelators such as protocatechuic acid (PCA) or catechol haven been shown to significantly improve the growth properties of *C. glutamicum* in minimal medium, that is reduction of the lag phase and constant exponential growth (Liebl et al., 1989). Consequently, PCA became a standard ingredient of the widely used CgXII minimal medium (Keilhauer et al., 1993; Unthan et al., 2014).

PCA (3,4‐dihydroxybenzoic acid) is a constitutional isomer of the more frequently employed siderophore precursor 2,3‐dihydroxybenzoic acid (2,3‐DHBA). Likewise, it can provide the functional moiety of such iron binding molecules (e.g. petrobactin) (Barbeau et al., 2002). A common response of many bacteria to iron limited conditions is the secretion of high affinity siderophores in order to make poorly soluble ferric iron (Fe^3+^) available. But also the catecholate compounds 2,3‐DHBA and PCA themselves were detected under iron limitation in the culture supernatants of *Bacillus subtilis*, *Paracoccus denitrificans* and *Bacillus anthracis*, respectively (Neilands, 1981; Peters & Warren, 1968; Tait, 1975), where they can increase the iron availability by chelation of Fe^3+^ and the chemical reduction to Fe^2+^ (Müller et al., 2020).

**FIGURE 1:**
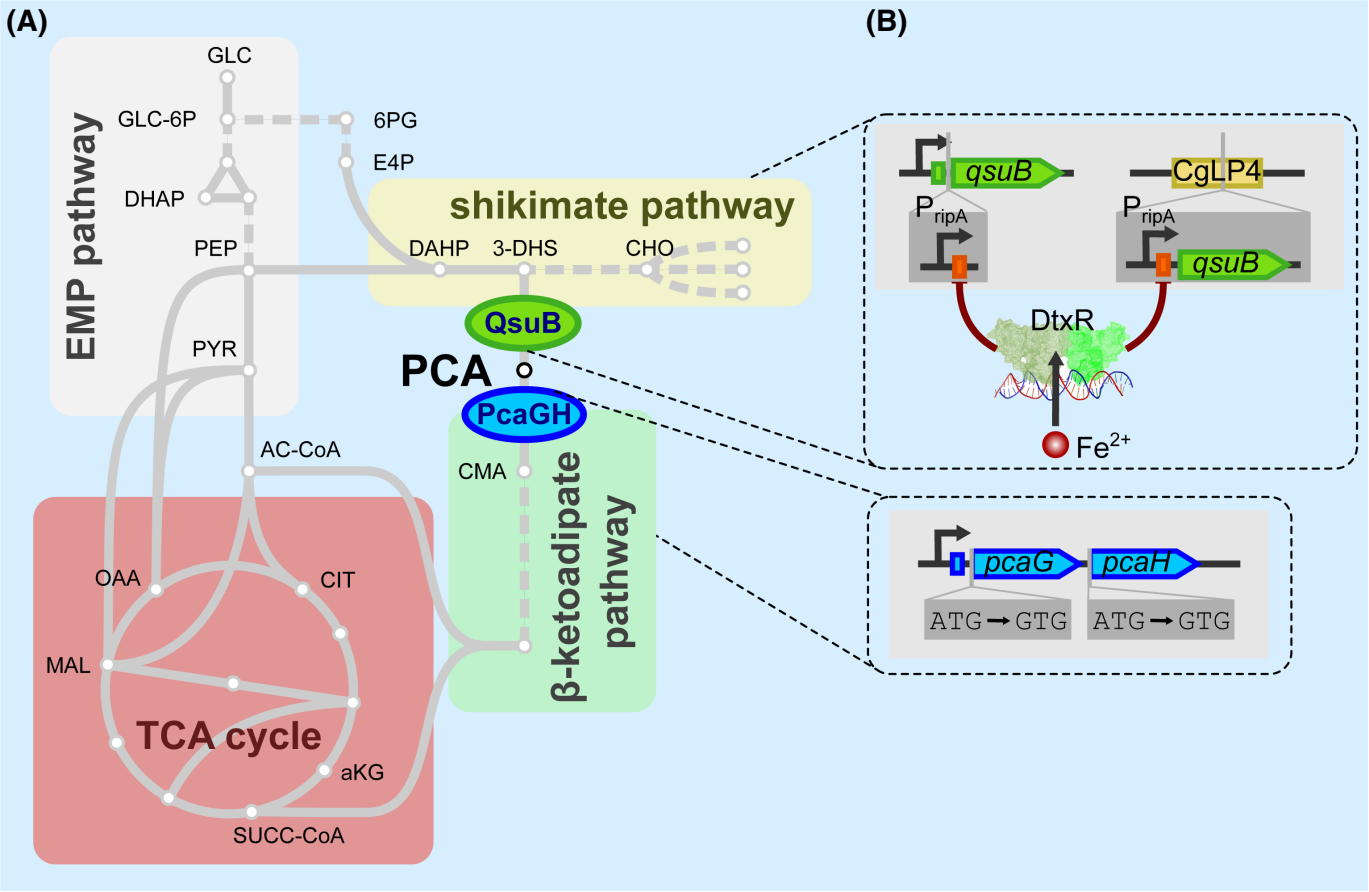
The central carbon metabolism of *C. glutamicum* including PCA biosynthesis via the shikimate pathway and its degradation via the β‐ketoadipate pathway (A). Metabolic engineering strategies to place the PCA synthesis under control of the master regulator of the iron homeostasis, DtxR (B): (i) replacement of the native *qsuB* expression control with the *ripA* promoter (P_ripA_), which is repressed by DtxR at Fe^2+^ excess, (ii) integration of an additional P_ripA_‐*qsuB* copy in the CgLP4 locus (Lange et al., 2017), (iii) increase of the PCA pool by mitigating expression of the *pcaG* and *pcaH* genes through the start codon exchange ATG → GTG. Abbreviations: 3‐DHS, 3‐dehydroshikimate; 6PG, 6‐phosphogluconate; AC‐CoA, acetyl‐CoA; CHO, chorismate; CMA, β‐carboxy‐*cis, cis*‐muconate; DAHP, 3‐desoxyarabinoheptulosanat‐7‐phosphate; E4P, erythrose‐4‐phosphate; GLC, glucose; GLC‐6P, glucose‐6‐phosphate; PCA, protocatechuic acid; PcaGH, PCA 3,4‐dioxygenase; PEP, phosphoenolpyruvate; PYR, pyruvate; QsuB, 3‐dehydroshikimate dehydratase; SUCC‐CoA, succinyl‐CoA.

The iron homeostasis of *C. glutamicum* was extensively studied with the focus on the transcriptional regulation, iron storage and mobilization, as well as on the utilization of alternative iron sources (Blombach et al., 2013; Brune et al., 2006; Follmann et al., 2009; Frunzke et al., 2011; Keppel et al., 2019; Küberl et al., 2016, 2020; Müller et al., 2020; Wennerhold et al., 2005; Wennerhold & Bott, 2006). The master regulator of iron homeostasis in *C. glutamicum* is DtxR which in response to the intracellular Fe^2+^ concentration controls the transcription of genes encoding proteins for iron acquisition, storage and mobilization as well as proteins responsible for iron–sulphur cluster assembly. Moreover, DtxR represses under iron excess the transcription of the *ripA* gene coding for the transcriptional regulator of iron proteins A (RipA). Under iron limitation, RipA represses the transcription of genes encoding enzymes such as aconitase or succinate dehydrogenase. So far, it is not clear, how iron is initially transported into the cells when *C. glutamicum* grows as monoculture, because siderophore biosynthetic genes were not identified in the genome although a large repertoire of siderophore uptake systems is available (Brune et al., 2006; Frunzke & Bott, 2008; Wennerhold & Bott, 2006). Interestingly, *C. glutamicum* is genetically equipped to form PCA, but unlike in other bacteria, PCA biosynthesis nor its degradation is regulated in response to the iron availability (Brune et al., 2006; Kalinowski et al., 2003; Shen et al., 2012; Wennerhold & Bott, 2006). During growth on glucose PCA is synthesized from 3‐dehydroshikimate, an intermediate of the shikimate pathway, catalysed by the 3‐dehydroshikimate dehydratase (QsuB) encoded by *qsuB* (Figure 1A). *C. glutamicum* also features the β‐ketoadipate pathway to utilize several aromatic compounds such as PCA as sole carbon and energy source (Kubota et al., 2014; Merkens et al., 2005; Shen & Liu, 2005; Teramoto et al., 2009; Zhao et al., 2010). PCA degradation is initiated by the PCA 3,4‐dioxygenase (PcaGH), which consists of two subunits encoded by *pcaG* and *pcaH* located in the *pcaHGBC* operon (Figure 1) (Zhao et al., 2010). Recently, we investigated biomass formation of the *C. glutamicum* WT in PCA‐deficient CgXII medium and observed a biphasic growth behaviour caused by a transient iron limitation (Müller et al., 2020). This growth retardation could be compensated by either the supplementation of PCA to the CgXII medium or by aeration of the bioreactor with an increased proportion of CO_2_ in the inlet air. It turned out that the presence of CO_2_/HCO_3_^−^ accelerates the chemical reduction of poorly soluble ferric iron (Fe^3+^) to biologically active ferrous iron (Fe^2+^) through phenolic acids and catechols including PCA (Müller et al., 2020).

To improve the growth properties of *C. glutamicum* in minimal medium without PCA supplementation, we installed an iron‐responsive PCA biosynthesis by exchange of the native *qsuB* promoter with the *ripA* promoter (P_ripA_), which is repressed by DtxR at Fe^2+^ excess, integrated of a second copy of P_ripA_‐*qsuB* into the genome and increased the PCA pool by mitigating expression of the *pcaG* and *pcaH* genes (Figure 1B).

## MATERIALS AND METHODS

### Bacterial strains, plasmids, primers and cultivation conditions

*Escherichia coli* DH5α was used as host for cloning applications (Hanahan, 1983). All *C. glutamicum* strains generated in this study were derivatives of the wild type ATCC 13032. Table 1 provides a list of all strains and plasmids used in this study. The primers are itemized in Table S1. Cryogenic stocks of all strains were prepared in 30% (v/v) glycerol and maintained at −80°C. Cell propagation during cloning was performed in complex medium (2x YT) containing 16 g Bacto‐tryptone, 10 g yeast extract and 10 g sodium chloride per L (Sambrook & Russell, 2001). Plates contained additionally 18 g agar L^−1^. When required a final concentration of 50 μg kanamycin mL^−1^ was added to the hand‐warm agar prior to pouring or to liquid media immediately before inoculation. Cultivation of *C. glutamicum* was performed in CgXII minimal medium (Table 2) with a starting of pH 7.4 (glucose) or 6.5 (acetate).

**TABLE 1:** Bacterial strains and plasmids.

Strain/plasmid	Relevant characteristics	Source/reference
Strains		
*E. coli* DH5α	*supE44* ∆*lacU169 (f80lacZ*∆*M15) hsdR17 recA1 endA1 gyrA96 thi relA1*	(Hanahan, 1983)
*C. glutamicum* WT	ATCC 13032—wildtype strain	(Abe et al., 1967) American Type Culture Collection
*C. glutamicum* FEM3	*C. glutamicum* ATCC 13032 (*cg3344‐cg3345*)'::P_ripA_ *‐lacI* (pJC4‐P_tac_‐*egfp*)	(Müller et al., 2020)
*C. glutamicum* P_ripA_‐*qsuB*	*C. glutamicum* WT with the promoter of the *ripA* gene controlling expression of the native *qsuB* gene	this study
*C. glutamicum* 2x (P_ripA_‐*qsuB*)	*C. glutamicum* P_ripA_‐*qsuB* with an additional copy of the P_ripA_‐*qsuB* cassette integrated in the CgLP4 locus	this study
*C. glutamicum* IRON+	*C. glutamicum* 2x (P_ripA_‐*qsuB*) with weakened PCA degradation through start codon exchanges (ATG → GTG) of the *pcaG* and *pcaH* genes	this study
*C. glutamicum* FEM3 IRON^+^	bioreporter strain *C. glutamicum* FEM3 containing the genetic modification of *C. glutamicum* IRON^+^	this study
Plasmids		
pK19*mobsacB*	plasmid for the markerless chromosomal integration/deletion in *C. glutamicum*, pMB1 oriV_*E. coli*_, oriT (RP4*mob*), *lacZα*, *sacB*_*B. subtilis*_, Kan^R^	(Schäfer et al., 1994)
pJC4‐P_tac_‐*egfp*	pJC4 derivative expressing the *egfp* gene (followed by the *rrnB* terminator) under control of the P_tac_ promoter, oriV_*E. coli*_, pCG1 oriV_*C. glutamicum*_, Kan^R^	(Müller et al., 2020)
pK19*mobsacB*‐∆*qsuB*::P_ripA_‐*qsuB*	pK19*mobsacB* derivative for the integration of the *ripA* promoter upstream of the native *qsuB* gene (*cg0902*)	this study
pK19*mobsacB*‐∆CgLP4::P_ripA_‐*qsuB*	pK19*mobsacB* derivative for the integration of a second P_ripA_‐qsuB cassette in the CgLP4 locus (Lange et al., 2017)	this study
pK19*mobsacB*‐*pcaGH* ^GTG^	pK19*mobsacB* derivative for the substitution of the native ATG start codon of the *pcaG* and *pcaH* genes with the weaker GTG start codon	this study

**TABLE 2:** Composition of CgXII medium used in this study.

Compound	Final concentration
(NH_4_)_2_SO_4_	5 g L^−1^
Urea	5 g L^−1^
MOPS buffer	21 g L^−1^
K_2_HPO_4_	1 g L^−1^
KH_2_PO_4_	1 g L^−1^
*Added from sterile stocks prior to inoculation*	
MgSO_4_	250 mg L^−1^
CaCl_2_	10 mg L^−1^
Biotin	0.2 mg L^−1^
FeSO_4_ × 7 H_2_O	16.4 mg L^−1^
MnSO_4_ × H_2_O	10 mg L^−1^
CuSO_4_ × 5 H_2_O	313 mg L^−1^
ZnSO_4_ × 7 H_2_O	1 mg L^−1^
NiCl_2_ × 6 H_2_O	0.02 mg L^−1^

*Note*: Salts were dissolved in VE‐H_2_O, pH adjusted to 7.4 (glucose) or 6.5 (acetate) with 5 M KOH and autoclaved. Trace elements were added from sterile 1000x concentrated stock solutions immediately before starting growth experiments. PCA was added from a sterile 1000x stock when indicated.

### Genetic modifications

DNA fragments for cloning were PCR amplified with the Q5 polymerase (New England Biolabs, Frankfurt, Germany) according to the manufacturers specifications. Agarose gel electrophoresis and standard techniques of molecular biology were performed as described by Sambrook & Russell, 2001. Isolation of chromosomal DNA of *C. glutamicum* WT, plasmid DNA and the purification of PCR products was carried out with the NucleoSpin Microbial DNA, NucleoSpin Plasmid and NucleoSpin Gel and PCR Clean‐up commercial kits (all purchased from MACHEREY‐NAGEL, Düren, Germany) in accordance with the manufacturer's instructions. Enzymes were obtained from New England Biolabs (Frankfurt, Germany) and oligonucleotides were supplied by Sigma Aldrich (Steinheim, Germany).

Plasmid pK19*mobsacB* was used for the chromosomal integrations in *C. glutamicum* based on selection/counter‐selection as described before (Schäfer et al., 1994). Briefly, since pK19*mobsacB* cannot be replicated in *C. glutamicum* WT, kanamycin resistance can only be acquired by integration of the entire plasmid through homologous recombination. In order to excise the vector backbone and retain the markerless integration, the counter‐selection is performed on sucrose containing agar plates. Only cells that eliminated the vector backbone with the *sacB* gene successfully can survive in this step. Cells carrying the desired genetic modification are finally verified by colony PCR (ColPCR) and discriminated from (re‐established) wild type cells and cells with mutations in the *sacB* promoter region. Derivatives of the pK19*mobsacB* carried typically 500 bp regions flanking the target locus (1500 bp for targeting the CgLP4 locus).

In order to place the native *qsuB* gene (*cg0502*) of *C. glutamicum* WT under control of the iron‐responsive transcriptional regulator DtxR, the promoter of the *ripA* gene was PCR amplified from the chromosomal DNA of *C. glutamicum* WT using the primer pair ripA1/ripA2. Since the intergenic region between *cg0501* and *cg0502* is only 23 bp long we integrated the P_ripA_ promoter upstream of the *qsuB* gene without removing the native genetic sequence in order to leave the coding regions intact. The 500 bp regions were PCR amplified from the chromosomal DNA with the primer pairs F1/F2 and qsuB1/qsuB2. All primers contained 20 bp overlaps with the anticipated neighbouring fragment required for homologous recombination. HindIII and BamHI linearized pK19*mobsacB* and all fragments were simultaneously joined by the isothermal Gibson assembly (Gibson, 2011; Gibson et al., 2009). All other plasmids were constructed accordingly. The 500 bp flanks for integration in the CgLP4 locus were amplified from the chromosomal DNA of *C. glutamicum* WT using the primer pairs CgLP4_1/CgLP4_2 and CgLP4_3/CgLP4_4, respectively. The integration cassette consisting of P_ripA_, qsuB and T_rrnB_ was amplified with the primer pairs ripA3/ripA2 and qsuB1/qsuB3 from the chromosomal DNA, and with TrrnB1/TrrnB2 using pFEM06 as templates. The native ATG start codons of *pcaG* and *pcaH* were simultaneously replaced by the weaker GTG through homologous recombination. Three fragments were amplified from the chromosomal DNA of *C. glutamicum* WT using the primer pairs F3/F4, pcaG1/pcaG2 and pcaH1/pcaH2, respectively, that encoded the desired GTG start codon (Table S1).

Competent cells of *E. coli* DH5α were transformed with the Gibson assembly mixes by electroporation (Dower et al., 1988). Positive transformants were screened by colony PCR and plasmids were finally verified by sequencing (Microsynth Seqlab, Göttingen, Germany). Competent cells of *C. glutamicum* were prepared according to Kirchner and Tauch (2003) and transformed with 500 ng of the purified plasmid by electroporation (van der Rest et al., 1999). Correct clones were identified by ColPCR or sequencing of the corresponding region to verify the start codon exchange.

### Shaking flask cultivations

Main growth experiments were all started from the same seed train precisely as described before (Müller et al., 2020). A cryogenic culture was streaked on 2x YT agar plates and incubated for 2 days at 30°C. A reaction tube containing 5 mL liquid 2x YT medium was inoculated with a single colony, grown over night (O/N) at 30°C shaking (180 rpm, shaking diameter 25 mm) and used completely to inoculate a 500 mL baffled shaking flask containing 50 mL 2x YT on the next day. After 8 h incubation shaking at 30°C, cells were harvested by centrifugation (10 min, 4000 × *g*) and the cell pellet was resuspended in 1 mL 9 g NaCl L^−1^. The OD_600_ was adjusted to 25 in order to reach a starting OD_600_ of 1 in the CgXII preculture by the addition of 2 mL inoculum. The preculture was again incubated O/N under identical conditions and the inoculum for the main experiments was prepared identically. Main cultures in CgXII minimal medium contained either 2% (w/v) glucose or 1% (w/v) acetate (as potassium acetate stock solution) and were supplemented with 30 mg PCA L^−1^ as indicated. Additional growth experiments were performed with about 0.3% (20 mM) PCA as the sole carbon and energy source. All cultures were incubated for 25 h (shaking flasks) under the same conditions as the precultures.

### Bioreactor cultivations

Bioreactor cultivations were operated in batch mode in four identical 2 L glass vessels (DASGIP®, Jülich, Germany) filled with 800 mL CgXII medium lacking MOPS buffer and urea. The pH of the cultivation medium was measured with a standard pH probe (405‐DPAS‐SC‐K8S/325, Mettler Toledo, Giessen, Germany) and maintained at 7.4 by the addition of 25% (v/v) ammonium hydroxide. Four different experiments were performed in parallel in order to compare the *C. glutamicum* WT and IRON^+^ strains aerated at 0.5 vvm with either CO_2_‐enriched or ambient air. Since the glass reactor could not be operated at overpressure, 30% CO_2_ was added to the inlet air in order to establish the same partial pressure of CO_2_ as before (Blombach et al., 2013; Müller et al., 2020). The stirrer speed was automatically controlled to realize dissolved oxygen concentrations >35%, which was monitored with a polarographic probe (Mettler Toledo, Giessen, Germany). The bioreactors were inoculated as described above and cultivated with 20 g glucose L^−1^ for 14 h at 30°C.

### BioLector cultivations

Microscale cultivations were performed in the BioLector device (m2p labs, Baesweiler, Germany) in 48‐well flower plates. Each well was filled with a total of 1 mL CgXII medium that was supplemented as indicated above. The inoculum was pre‐diluted in order to keep the total volume identical. The cultivation plate was sealed with an air‐permeable membrane and incubated at 30°C at a shaking frequency of 1000 rpm. In order to monitor the cell density, the backscatter value at 620 nm was automatically measured every 10 min.

### Monitoring growth and determination of the biomass concentration

The cell density was monitored during shaking flask and bioreactor cultivations by measuring the optical density at 600 nm (OD_600_). Biomass concentrations (as cell dry weight (CDW) per L) were calculated by the correlation factor, which was specific for the spectrophotometer (Ultrospec 10 cell density meter, Harvard Biochrom, Holliston, MA, USA): c_CDW_ = OD_600_ × 0.26 g L^−1^. For an accurate determination of the biomass at the end of the bioreactor cultivations 50 mL culture broth was harvested (10 min, 4000 × *g*, room temperature (RT)), washed three times with 20 mL 0.9 g NaCl L^−1^, resuspended in 10 mL fully demineralized H_2_O and transferred to a pre‐weighed 50 mL glass beaker. The beakers were dried for 48 h in a static incubator at 105°C until all humidity was evaporated. Finally, the biomass was weighed out on an analytical balance.

### Quantification of sugars, alcohols and organic acids.

The consumption of glucose during bioreactor cultivations and the potential accumulation of acetate, lactate, succinate, formate and ethanol was monitored by HPLC‐RID analysis as described previously (Siebert et al., 2021). Metabolites were denoted as ‘not detectable’, when peak areas were smaller than the quantification limit of 1 mM. The presence of PCA in culture supernatants was monitored by HPLC‐DAD (Siebert et al., 2021) with a quantification limit of 1 μM.

### Fluorescence experiments

In order to monitor differences in the intracellular Fe^2+^ concentration, fluorescence experiments were performed with *C. glutamicum* FEM3‐derived reporter strains (Müller et al., 2020) after 25 h of cultivation. Suspensions were diluted to an OD_600_ of approximately 11 with NaCl solution (9 g L^−1^). 100 μL of the dilution was transferred into one cavity of a 96‐well microtiter plate and the fluorescence was measured using a plate reader (Tecan Spark Multimode Microplate reader, Tecan, Zürich, Switzerland) as technical duplicates at the following conditions: excitation wavelength: 485 ± 20 nm, emission wavelength: 535 ± 20 nm, gain: 87. Background fluorescence of culture supernatants was subtracted from the fluorescence of the cell suspension due to the autofluorescence of the CgXII medium and the data was normalized to the biomass concentration as before (Müller et al., 2020).

### Calculation of μ, Y_X/S_ and time shift of exponential growth phases

Growth rates (μ) were determined by linear regression and least square fitting of the logarithmized cell density during the exponential growth phase. Biomass yields per substrate (Y_X/S_) were calculated from the experimental data of final biomass concentration and initial glucose concentration of bioreactor cultivations once all glucose was exhausted.

Different growth properties of the engineered strains were quantified by calculating the time shift of the exponential growth phases (∆t_exp_). In BioLector experiments the cultivation time for reaching a backscatter value in the mid exponential growth phase (800) was extracted from the data. The ∆t_exp_ was calculated for each cultivation by subtracting the cultivation time of the WT + PCA. In shaking flask experiments, the cultivation time until reaching a biomass concentration of 6 g_CDW_ L^−1^ was calculated from the exponential growth curve. The differences between the strains and cultivation conditions were calculated as before. Standard deviations of all ∆t_exp_ values were calculated by Gaussian error propagation.

### Statistics

All experiments were performed at least as three independent biological replicates with individual seed trains starting from different colonies on the agar plates. If not stated otherwise, data represent mean values with error bars representing the standard deviation of *n* ≥ 3. When necessary, standard deviations were calculated by Gaussian error propagation. Significant differences of the normalized fluorescence data were analysed by a two sample Student's *t*‐test assuming equal variances and the significance levels indicated as *, *p* < 0.05, **, *p* < 0.01, ***, *p* < 0.001.

## RESULTS

### Engineering *C. glutamicum* to improve the intracellular iron availability

In PCA‐deficient CgXII medium with glucose we previously observed a transient iron limitation of *C. glutamicum* WT, which results in a growth retardation compared to medium with externally supplied PCA (Müller et al., 2020). *C. glutamicum* ATCC 13032 is genetically equipped to synthesize PCA on its own, but unlike in other bacteria, the endogenous pathway is not regulated in response to the iron availability (Brune et al., 2006; Teramoto et al., 2009; Wennerhold & Bott, 2006). We hypothesized that the intracellular Fe^2+^ availability and thus the growth properties in non‐PCA‐supplemented cultures could be increased, if the *qsuB* gene, which is responsible for PCA formation was part of the DtxR regulon, the master regulator of iron homeostasis. Consequently, we placed the expression of the native *qsuB* gene under control of the *ripA* promoter, which is in turn only regulated by DtxR. The resulting strain should reveal a higher *qsuB* expression and thus PCA synthesis than *C. glutamicum* WT when the intracellular Fe^2+^ availability is low. High Fe^2+^ levels would induce a DtxR mediated repression of the *qsuB* expression to avoid prodigal carbon flux.

**FIGURE 2:**
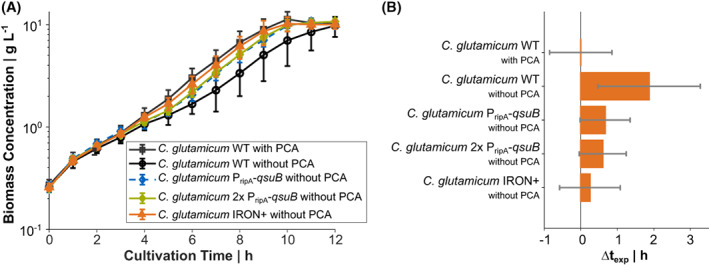
Shaking flask cultivation of *C. glutamicum* WT and engineered derivatives in CgXII minimal medium containing 2% (w/v) glucose and PCA as indicated. Growth curve over time (A) and time shifts of the exponential growth curves with regard to the *C. glutamicum* WT cultivation with PCA as reference (B). All data points represent mean values with error bars indicating the standard deviation of *n* ≥ 3. Standard deviation in (B) was calculated by error propagation.

In CgXII medium supplemented with 2% (w/v) glucose without PCA, *C. glutamicum* WT showed biphasic growth with a growth rate of 0.38 ± 0.02 h^−1^ in the second of the two growth phases, whereas the supplementation of PCA resulted in constant exponential growth throughout with a rate of 0.41 ± 0.02 h^−1^ (Figure 2A). Compared to PCA‐supplemented medium the onset of exponential growth (∆t_exp_) was delayed by about 1.86 ± 1.40 h when PCA was absent (Figure 2B). In contrast, *C. glutamicum* harbouring the synthetic P_ripA_‐*qsuB* element recovered quicker from the transient iron limitation as the WT and showed an improved μ of 0.41 ± 0.02 h^−1^ in the second growth phase (Figure 2A). However, WT‐like growth with PCA could not be fully restored and *C. glutamicum* P_ripA_‐*qsuB* still exhibited a ∆t_exp_ of 0.67 ± 0.68 h (Figure 2B). Therefore, we integrated a second copy of the P_ripA_‐*qsuB* cassette into the CgLP4 locus (Lange et al., 2017) of *C. glutamicum*. In the absence of PCA, the resulting strain *C. glutamicum* 2x P_ripA_‐*qsuB* showed the same μ and a slightly reduced ∆t_exp_ compared to the parental strain with one copy of the P_ripA_‐*qsuB* cassette (Figure 2A,B). We reasoned that the PCA pool size is not solely determined by the expression strength of *qsuB*, but also by PCA degradation (Figure 1). To slow down the first step of the β‐ketoadipate pathway, we additionally replaced the ATG start codon of the *pcaG* and *pcaH* genes, which encode two subunits of the protocatechuate 3,4‐dioxygenase with the weaker GTG start codon in *C. glutamicum* 2x P_ripA_‐*qsuB*. The resulting strain *C. glutamicum* IRON+ could grow on PCA as sole carbon and energy source. However, its growth rate was reduced by 37% (μ = 0.26 ± 0.01 h^−1^ compared to μ = 0.41 ± 0.06 h^−1^ of the WT strain; data not shown). During the cultivation with 2% (w/v) glucose, this strain still grew at μ = 0.41 ± 0.02 h^−1^ during the exponential phase without PCA and showed an almost identical growth pattern compared to *C. glutamicum* WT cultivated in the presence of PCA with an average ∆t_exp_ of only 0.25 ± 0.83 h (Figure 2A,B). In the applied shaking flask system all engineered strains reached the same biomass concentration at the end of the cultivation and neither showed an improved growth property compared to the WT in the presence of PCA nor secreted PCA into the culture supernatant in concentrations higher than 1 μM (quantification limit; data not shown).

**FIGURE 3:**
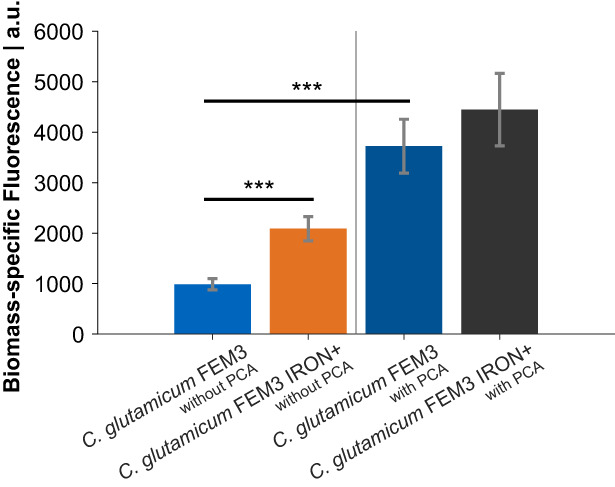
Biomass‐specific fluorescence after 25 h cultivation in shaking flasks containing CgXII minimal medium, 2% (w/v) glucose and PCA as indicated. Genetic modifications of *C. glutamicum* IRON+ were re‐engineered in the reporter strain background *C. glutamicum* FEM3 (Müller et al., 2020). Bars represent mean values with error bars indicating the standard deviation of *n* = 5. Significance levels indicated as *, *p* < 0.05, **, *p* < 0.01, ***, *p* < 0.001.

In order to investigate the impact of the introduced genetic modifications on the intracellular Fe^2+^ availability, we re‐engineered the genetic modifications of *C. glutamicum* IRON+ in the reporter strain background of *C. glutamicum* FEM3. In this strain high Fe^2+^ concentrations induce the binding of DtxR to P_*ripA*_, thus provoking repression of *lacI*, which eventually results in *egfp* expression under the control of the strong *tac* promoter (P_*tac*_) (Müller et al., 2020). The biomass‐specific fluorescence of *C. glutamicum* FEM3 IRON+ after 25 h of cultivation in CgXII medium without PCA was more than twice as high as that of the basic reporter strain *C. glutamicum* FEM3 (Figure 3), indicating significantly increased intracellular Fe^2+^ levels (*p*‐value = 1.17 × 10^−4^) as a result of the introduced genetic modifications. However, the metabolic engineering approach to increase the carbon flux towards PCA could not completely replace the external addition of PCA, which provoked highest biomass‐specific fluorescence in both strains (Figure 3).

### Bioreactor cultivations with *C. glutamicum* IRON+

**FIGURE 4:**
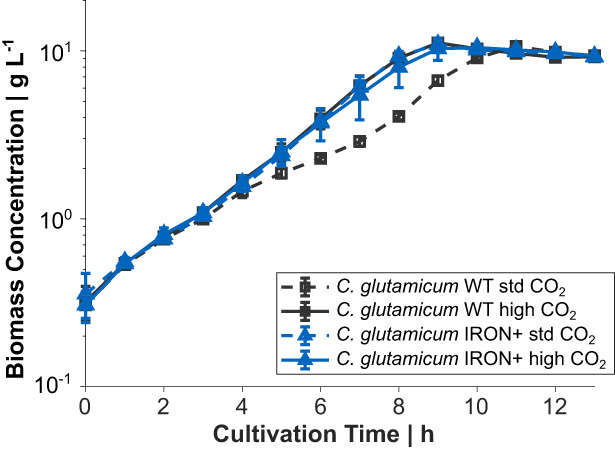
Bioreactor fermentation of *C. glutamicum* WT and *C. glutamicum* IRON+ with ambient air and 30% (v/v) CO_2_ in the inlet air, respectively. Growth curves represent mean values with error bars indicating the standard deviation of *n* = 3.

Next, we analysed the growth performance of *C. glutamicum* IRON+ in bioreactor cultivations in CgXII medium with 2% (w/v) glucose in the absence of PCA. As observed before (Müller et al., 2020), when aerated with pressurized air with 0.04% CO_2_ the *C. glutamicum* WT showed biphasic growth with a μ of 0.34 ± 0.02 h^−1^ in phase two and exhibited improved and constant growth with a μ of 0.41 ± 0.02 h^−1^ when the inlet air was enriched with 30% CO_2_ (Figure 4). When the newly constructed strain *C. glutamicum* IRON+ was cultivated, we could not observe such a growth retardation under both conditions. *C. glutamicum* IRON+ grew under standard conditions (pressurized air) and with CO_2_ enriched air exponentially throughout the cultivation with a μ of 0. 41 ± 0.01 h^−1^, which is identical to the cultivation of the WT with 30% CO_2_ (Figure 4).

### Growth properties of *C. glutamicum* IRON+ at small inoculum sizes

To achieve reliable and reproducible growth with *C. glutamicum* in PCA‐free CgXII medium, a relatively high starting biomass concentration of at least 0.3 g_CDW_ L^−1^ is commonly applied. Unthan et al. (2014) showed that PCA supplementation also ensured constant growth with smaller inoculum size. Consequently, we analysed the effect of a decreasing initial cell density on the course of the cultivation. Microscale cultivations were performed in the BioLector system that allows to continuously monitor the cell density via the backscatter signal. In this system we compared the effect of different starting ODs (OD^start^ = 1, which corresponds to 0.3 g_CDW_ L^−1^, OD^start^ = 0.2 and 0.02) on growth of *C. glutamicum* WT and *C. glutamicum* IRON+.

**FIGURE 5:**
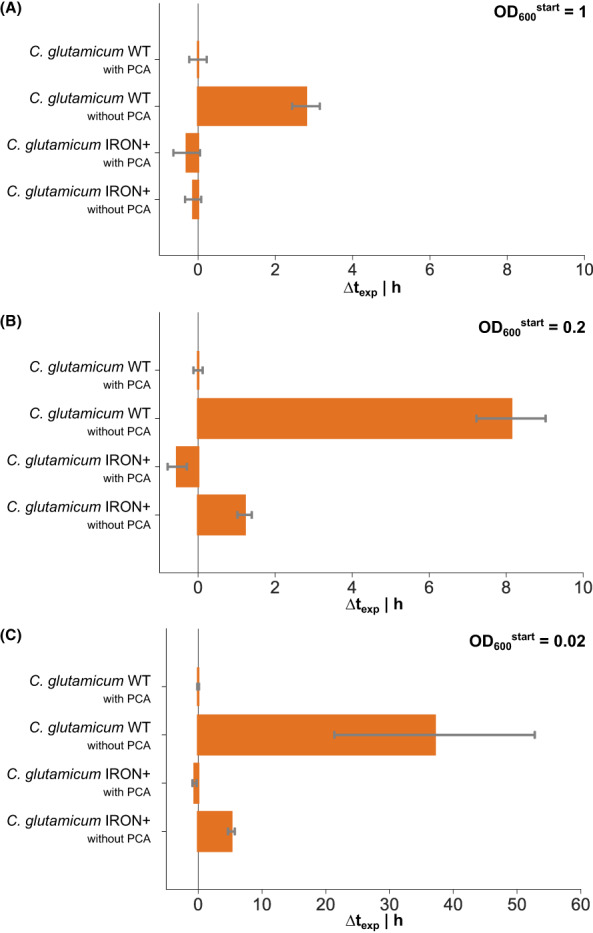
Microscale cultivations (BioLector) of *C. glutamicum* WT and *C. glutamicum* IRON+ in CgXII minimal medium containing 2% (w/v) glucose and PCA as indicated. Bars represent the time shifts (∆t_exp_) of the exponential growth curves with regard to the *C. glutamicum* WT cultivation with PCA as reference, starting the cultivation at an initial optical density of OD = 1 (A), OD = 0.2 (B) and OD = 0.02 (C). All data points represent mean values with error bars indicating the standard deviation of *n* ≥ 5. The standard deviation was calculated by error propagation.

When PCA was supplemented in cultivations with a OD^start^ = 1, *C. glutamicum* WT grew at μ = 0.52 ± 0.01 h^−1^, and the biomass concentration peaked between 8 and 9 h (Figure S1). We compared the shift of exponential growth phases at lower OD^start^ as before. Since backscatter values are not accurately proportional at low OD_600_ we calculated the theoretical shift of exponential growth phases if cultivations did not experience a lag phase. Assuming a constant μ = 0.52 h^−1^ independent of the inoculum density, exponential growth phases should be shifted theoretically by 3.1 h when the cultivation is started from OD^start^ = 0.2 instead of 1. A further reduction of the OD^start^ by the factor of 10 would result in a shift by 7.5 h compared to OD^start^ = 1. The actual time shifts of 3.4 ± 0.2 and 7.6 ± 0.2 h for cultivations of *C. glutamicum* WT in the presence of PCA with an OD^start^ = 0.2 and 0.02 are thus in good agreement with the expectations (Figure 5A–C), indicating that growth is not limited by the lower inoculum sizes as long as PCA is supplemented. As before, the exponential growth phase of *C. glutamicum* WT in a non‐PCA‐supplemented culture was significantly retarded in comparison with PCA‐supplemented cultivations (Figure S2). ∆t_exp_ increased from 2.8 ± 0.4 h at an OD^start^ = 1 to 8.1 ± 0.9 and 37.1 ± 15.7 h at an OD^start^ of 0.2 and 0.02, respectively (Figure 5A–C). In contrast, *C. glutamicum* IRON+ showed an improved performance at low OD^start^. Compared to the reference condition (*C. glutamicum* WT in PCA‐supplemented medium), the ∆t_exp_ was shifted by only 1.2 ± 0.2 h at OD^start^ = 0.2 and 5.2 ± 0.5 h at OD^start^ = 0.02, which is a reduction of the growth retardation by 85% and 86%, respectively. At starting OD_600_ = 1 a delay of *C. glutamicum* IRON+ compared to the PCA‐supplemented WT cultivation was completely vanished.

### Growth properties of *C. glutamicum* IRON+ on acetate

**FIGURE 6:**
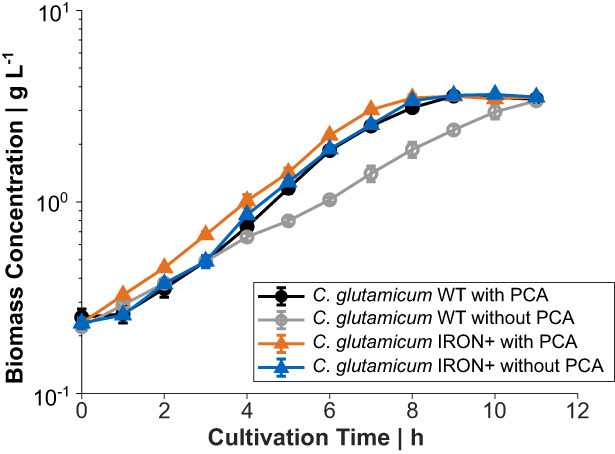
Shaking flask cultivation of *C. glutamicum* WT and *C. glutamicum* IRON+ in CgXII minimal medium containing 1% (w/v) acetate and PCA as indicated. Data points of the growth curve over time represent mean values with error bars indicating the standard deviation of *n* = 3.

Then, we characterized the growth of *C. glutamicum* IRON+ on acetate as an example of a gluconeogenic substrate. Similar to the cultivations on glucose, growth of *C. glutamicum* WT on acetate ceased about 2 h later, when PCA was not supplemented in the cultivation medium (Figure 6). Interestingly, the exponential growth rate (0.26 ± 0.01 h^−1^) was reduced by about one third compared to the PCA‐supplemented condition (μ = 0.39 ± 0.01 h^−1^). Such prominent differences of the exponential growth rate were not noted when using glucose as the carbon source. The engineered strain IRON+ did not reveal a growth retardation in any cultivation condition and grew at a similar rate as the PCA‐supplemented WT (μ = 0.37 ± 0.00 h^−1^ (without PCA) and 0.38 ± 0.00 h^−1^). It was interesting to note, that IRON+ initiated growth on acetate quicker than in any other conditions when PCA was supplemented.

## DISCUSSION

Although genetically equipped for the biosynthesis of PCA, *C. glutamicum* does not regulate the expression of the responsible genes in response to the intracellular iron availability (Brune et al., 2006; Wennerhold & Bott, 2006). We have recently shown, that *C. glutamicum* WT experiences a transient iron limitation in CgXII medium lacking PCA, which results in a growth delay during the initial phase of the cultivation (Müller et al., 2020). The external supplementation of PCA in CgXII minimal medium is a commonly employed strategy (Keilhauer et al., 1993), but might be undesired because of being expensive and providing an additional carbon source (Graf et al., 2019; Shen et al., 2012). In this study we artificially ushered the control of *qsuB* expression into the iron‐responsive DtxR regulon. This approach was inspired by nature, reflecting that other organisms regulate their biosynthesis of PCA or the structural analogue 2,3‐DHBA in response to the intracellular iron availability in turn (Garner et al., 2004; Neilands, 1981; Peters & Warren, 1968; Tait, 1975). We replaced the native promoter of the *qsuB* gene by P_ripA_ and introduced a second copy of the P_ripA_‐*qsuB* cassette into the genome of *C. glutamicum*, which indeed improved the growth properties but not to the level of PCA‐supplemented cultures. It was necessary to additionally reduce the degradation of PCA in order to enhance the growth phenotype further. This step is plausible since *C. glutamicum* possesses a functional β‐ketoadipate pathway and can efficiently utilize several aromatic compounds such as PCA (Shen et al., 2012). Accordingly, Okai et al. (2017) and Kallscheuer and Marienhagen (2018) engineered *C. glutamicum* for the production of hydroxybenzoic acids and showed the necessity of an inactive degradation of PCA for efficient overproduction of this aromatic compound. With our previously designed iron reporter strain *C. glutamicum* FEM3 (Müller et al., 2020) we show, that the improved growth properties in minimal medium caused by the introduced genetic modifications correlates with an increased intracellular Fe^2+^ availability.

Interestingly, the beneficial growth properties of *C. glutamicum* IRON+ could also be exploited when acetate was used as sole carbon and energy source. Recently, Graf et al. (2019) evolved a fast‐growing variant of *C. glutamicum* WT (designated as EVO5), which proliferates independently of PCA. Genome re‐sequencing of *C. glutamicum* EVO5 identified overall 10 mutations with three mutations located in the genes coding for the transcriptional regulators DtxR, RipA and RamA. Re‐engineering of the *ramA* mutation in *C. glutamicum* WT improved the growth rate in PCA‐deficient minimal medium on glucose, however, led to significantly impaired growth on acetate (Graf et al., 2019). In contrast, *C. glutamicum* IRON+ showed also in CgXII medium with acetate improved growth properties. We could not simply compensate the transient iron limitation during the initial growth phase on acetate with *C. glutamicum* IRON+ but the growth rate was 42% higher than that of the WT during the entire cultivation. A reduction of the growth rate has been reported previously (Wendisch et al., 2000) and might be due to an uncoupled membrane potential during growth on weak acids (Axe & Bailey, 1995; Baronofsky et al., 1984; Kiefer et al., 2020). In this context it is not clear, whether PCA can alleviate the growth limiting stress conditions through its inherent redox chemistry (i.e. acting as an electron shuttle, Perron & Brumaghim, 2009) or whether the enzymatic stress‐response benefits from the increased Fe^2+^ availability of the strain. Given the fact, that acetate gains increasing importance as an alternative carbon and energy source for microbial production processes (Kiefer et al., 2020; Merkel et al., 2022; Schmollack et al., 2023), future research needs to address this effect as well as the performance of *C. glutamicum* IRON+ when exposed to different environmental stresses.

Although the *qsuB* gene is monocistronically transcribed in ATCC 13032 (Pfeifer‐Sancar et al., 2013) it is located in a gene cluster with *qsuC* and *qsuD* encoding dehydroquinate dehydratase and quinate/shikimate dehydrogenase, respectively. Consequently, the replacement of the native promoter by P_ripA_ might have increased *qsuCD* expression, too, which could additionally increase carbon flux towards the QsuB substrate 3‐dehydroshikimate (Kubota et al., 2014; Teramoto et al., 2009). Notably, not only PCA has a growth promoting effect. Also (di‐)phenolic compounds, such as catechol (Liebl et al., 1989), ferulic acid and vanillin facilitate growth of *C. glutamicum* (Siebert et al., 2021). We showed that functionalized aromatic compounds, with a mix of amino and hydroxyl groups or two adjacent hydroxyl groups chelate iron and/or reduce Fe^3+^, which improves the overall intracellular iron availability (Müller et al., 2020). And even indole was found to reduce extracellular Fe^3+^ (Walter et al., 2020). Therefore, when feedstocks such as lignocellulosic hydrolysates, which contain diphenolic compounds, are supplemented to the minimal medium or strains, which overproduce such molecules are utilized (Kallscheuer & Marienhagen, 2018; Kim et al., 2022; Okai et al., 2016, 2017), the application of *C. glutamicum* IRON+ might not be required. The fact that the soil bacterium *C. glutamicum* naturally encounters this substrate mixture might also explain the greater iron availability in its natural habitat, and why *qsuB* expression is not evolutionarily placed under control of the iron homeostasis regulators. However, the iron acquisition mode of *C. glutamicum* is not known, yet. An interesting future research objective is to differentiate whether the entire Fe^3+^‐chelates are internalized by *C. glutamicum* prior to the release of Fe^2+^ (i.e. via one of the siderophore‐specific transport systems) or whether the chemical reduction takes place spontaneously in the extracellular environment and iron is then taken up via Fe^2+^‐specific transport proteins that are also annotated in the genome (Frunzke & Bott, 2008).

The final strain *C. glutamicum* IRON+ performs well at different cultivation scales, does not accumulate quantifiable levels of PCA in the culture supernatant and maintains an equal Y_X/S_ as the WT. By that, *C. glutamicum* IRON+ features an interesting genetic basis that could be further engineered for production purposes and represents a neat host strain for laborious screening approaches, as well as large‐scale fermentations. Moreover, the introduced genetic modifications might be combined with the engineered biotin prototrophic *C. glutamicum* strains (Ikeda et al., 2013; Peters‐Wendisch et al., 2014) to obtain a prototrophic platform strain, which provides reliable growth even at low initial biomass concentrations.

## CONCLUSION

This study demonstrates the successful re‐organization of transcriptional control by applying a naturally inspired approach. *C. glutamicum* might not encounter iron restricted conditions in its natural habitat, but does so in monoseptical cultivations. Consequently, by controlling the expression of the endogenous PCA synthesis gene *qsuB* in response to the iron availability and by mitigating its degradation, we ended up with a strain that exhibits a significantly higher intracellular Fe^2+^ concentration, superior growth properties on different substrates and retains an identically high biomass yield as the WT. Hence, *C. glutamicum* IRON+ represents an interesting platform for further engineering approaches in an academic as well as industrial environment, because it overcomes the need of undesirable PCA supplementation, which is expensive on the one hand and provides another carbon source on the other.

## AUTHOR CONTRIBUTIONS

**Felix Sebastian Max Thoma:** Conceptualization (lead); investigation (lead); methodology (lead); writing – original draft (lead); writing – review and editing (lead). **Christof Appel:** Investigation (supporting). **Dominik Russ:** Investigation (equal); methodology (equal). **Janine Huber:** Investigation (supporting). **Felix Werner:** Investigation (supporting). **Bastian Blombach:** Conceptualization (lead); funding acquisition (lead); project administration (lead); resources (lead); supervision (lead); writing – original draft (lead); writing – review and editing (lead).

## FUNDING INFORMATION

No funding information provided.

## CONFLICT OF INTEREST STATEMENT

The authors declare that there are no competing interests associated with this work.

## Supporting information


Figure S1.
Figure S2.
Table S1.
