# Supplementary material for: Improving growth properties of Corynebacterium glutamicum by implementing an iron‐responsive protocatechuate biosynthesis
Source: Microb Biotechnol. 2023 Mar 11;16(5):1041–53. doi: 10.1111/1751-7915.14244 (PMC10128138; doi:10.1111/1751-7915.14244)
Supplement: Supplementary file 1 — Figure S1. Figure S2. Table S1. [file MBT2-16-1041-s001.docx]

**Supplementary material**

***Supplementary Table***

**Table S1:** List of Primers. The desired weak start codon GTG was encoded on the primers (wrong priming) and is shown in bold.

| **Name** | **Sequence (5’ 🡪 3’)** |
| --- | --- |
| ripA1 | GGAATCTTTGACCACCGCAGCGACCTGC |
| ripA2 | ATCTCATCCTCACTACAAGC |
| F1 | AACAGCTATGACCATGATTACGCCAAGCTTATTGGTCGCCGACC |
| F2 | GCAACTTTGCGAGCAGGTCGCTGCGGTGGTCAAAGATTCCTTTTGG |
| qsuB1 | AATTTGCTTGTAGTGAGGATGAGATATGCGTACATCCATTGCC |
| qsuB2 | AGTGAATTCGAGCTCGGTACCCGGGGATCCAACGTATCCAAGCAGGTTC |
| CgLP4_1 | AACAGCTATGACCATGATTACGCC AAGCTT CCGTTCGGCTGACTC |
| CgLP4_2 | GCAACTTTGCGAGCAGGTCGCTGCG CATCAAAAAATCCGCCGTTC |
| ripA3 | ACAAGGAACGGCGGATTTTTTGATG CGCAGCGACCTGC |
| qsuB3 | CTCTCATCCGCCAAAACAGCCAAGC CTAGTTTGGGATTCCCCG |
| TrrnB1 | GCTTGGCTGTTTTGGC |
| TrrnB2 | CAGGAGAGCGTTCACC |
| CgLP4_3 | GTCGGTGAACGCTCTCCTGACTAAGTGAGTTTGGATGCG |
| CgLP4_4 | AGTGAATTCGAGCTCGGTACCCGG GGATCC CTCACTAGTACGCGGATAAATG |
| F3 | AACAGCTATGACCATGATTACGCCAAGCTT GGGACGGCACCTG |
| F4 | GGATGTC**CAC**GCGAGACCTTTCTGCG |
| pcaG1 | GTCTCGC**GTG**GACATCCCACACTTCGC |
| pcaG2 | TATCAAT**CAC**TGCAAAACCCTTTACTCAAATGG |
| pcaH1 | GGGTTTTGCA**GTG**ATTGATACAGGGAAGAACGG |
| pcaH2 | AGTGAATTCGAGCTCGGTACCCGGGGATCC ACAGCCAAAACTGGGTC |

***Supplementary Figures***


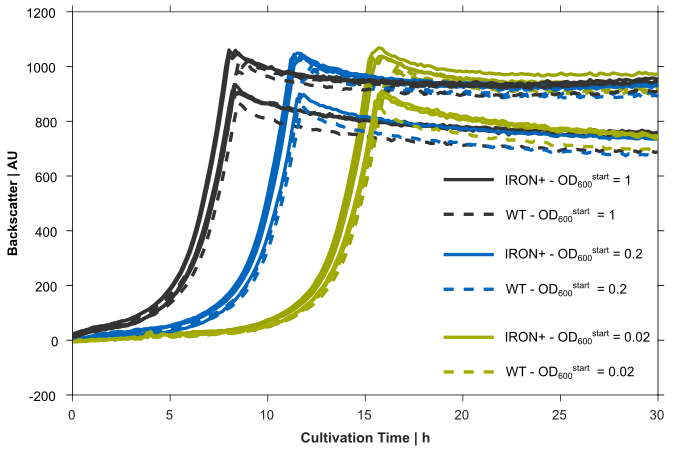
 **Supplementary Figure 1:** BioLector cultivation of *C. glutamicum* WT and *C. glutamicum* IRON+ in PCA-supplemented CgXII medium (2% (w/v) glucose) inoculated with different starting OD_600_.

**
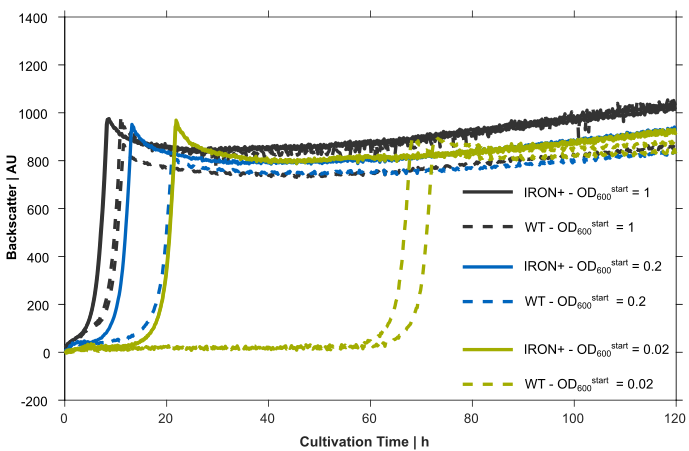
**

**Supplementary Figure 2:** BioLector cultivation of *C. glutamicum* WT and *C. glutamicum* IRON+ in PCA-free CgXII medium (2% (w/v) glucose) inoculated with different starting OD_600_.
